# Intentions of preventive depression management for a healthy pregnancy by gender among unmarried college students in Korea: a cross-sectional study

**DOI:** 10.1186/s12978-022-01380-3

**Published:** 2022-03-24

**Authors:** Hae Won Kim, Jieun Kim, Saem Yi Kang

**Affiliations:** 1 Research Institute of Nursing Science, Center for Human-Caring Nurse Leaders for the Future by Brain Korea 21 (BK 21) Four Project, College of Nursing, Seoul National University, Seoul, Republic of Korea; 2 Red Cross College of Nursing, Chung-Ang University, Seoul, Republic of Korea; 3 College of Nursing, Institute of Health Sciences, Gyeongsang National University, 816-15 Jinju-daero, Jinju, 52727 Republic of Korea

**Keywords:** Depression, Gender, Mental health, Reproductive health, Self-efficacy

## Abstract

**Background:**

Early adulthood is a significant period for preventive depression management for a healthy pregnancy. However, previous public health initiatives have not yet emphasized preparation for a healthy pregnancy in this population. In addition, pregnancy planning has traditionally been regarded as women’s responsibility, so intervention strategies may differ by gender. This study explored intentions of preventive depression management for a healthy pregnancy among unmarried college students, as well as factors influencing those intentions, by applying the Theory of Planned Behavior (TPB) model.

**Methods:**

For this cross-sectional survey, 828 unmarried college students aged 18–29 were recruited from a national university in Korea from July to September 2019. The chi-square test and t-test were used to compare gender differences in general characteristics, the current level of depression, and constructs of the extended TPB. Hierarchical regression was performed to identify factors influencing the intention of preventive depression management for a healthy pregnancy.

**Results:**

The intention to manage depression was significantly higher in men than in women (*t* = 2.36, *p* = 0.019). The factors affecting the intention of preventive depression management for a healthy pregnancy were components of TPB in both women and men, of which self-efficacy had the greatest effect (female: *β* = 0.34, *p* < 0.001, male: *β* = 0.30, *p* < 0.001). The current level of depression was a significant factor for women only (*β* = 0.10, *p* = 0.014).

**Conclusions:**

Our study results underscore the need for nurses to perform preventive interventions and provide directions to develop interventions to improve the intention of depression management for unmarried college students. It is necessary to intervene with all the constructs of the TPB, especially self-efficacy, for both men and women. It is also important to check and address the current depression status of unmarried female college students.

## Plain English summary

Starting in early adulthood, such as college students in the preconception stage, it is necessary to take care of one’s mental health (including depression) in order to have a healthy pregnancy in the future. The early identification of adults needing depression management is also a major challenge. In this study, an online survey was conducted to investigate levels of depression among Korean college students and their intention to manage depression for a healthy future pregnancy, as well as to identify the influencing factors.

Convenience recruitment was conducted by e-mail at one university in Korea, and 828 male and female college students responded to the survey. An analysis of the factors affecting the intention to manage depression for a healthy future pregnancy identified the components of the Theory of Planned Behavior as influencing factors in both men and women. In particular, self-efficacy had the greatest influence. However, the current level of depression was only a significant factor among women.

These findings could help nurses recognize differences in gender characteristics for the intention of preventive depression management for a healthy pregnancy, and provide evidence to develop and implement the intervention. These efforts will ultimately reduce high-risk pregnancies due to poor mental health.

## Background

The Centers for Disease Control and Prevention (CDC) suggest that “getting mentally healthy” should be a common component of preparation for a healthy pregnancy in the pre-pregnancy stage [1]. Most college students are in the preconception stage [2], and are particularly at risk for mood disorders because of financial difficulties such as tuition and living expenses, increased academic demands, forming new social relationships, making changes in health behavior, loss of identity, the stressful tasks of separation and individuation from their family, and the major life transition to the workforce [3, 4]. In pregnant women, mood disorders increase the risk of premature delivery, the difficulty of breastfeeding initiation, and emotional and language development problems in children [4–6]. Having a strategy for depression prevention makes it easier to transition to depression management behavior, which can reduce college students’ depression and ultimately decrease the occurrence of perinatal depression.

In addition, the preconception period has become longer with global trends for increasing age at conception, despite increasing adolescent pregnancy rates, so preventive health care is essential. South Korea has the highest mean maternal age among all Organization for Economic Co-operation and Development (OECD) countries, as well as the fastest rate of increase in maternal age [7], underscoring the importance of planning for a healthy pregnancy. Nonetheless, it has proven difficult to reinforce depression management behavior among college students who are not planning to become pregnant because public health has not yet placed a sufficient emphasis on preparation for a healthy pregnancy [2]. This study attempted to take a preventive perspective regarding approaches to managing depression related to pregnancy.

According to the expanding Theory of Planned Behavior (TPB), intention strongly predicts actual behavior [8], and previous research confirmed that the intention of preparing healthy pregnancy in college students predicted the corresponding behavior [9]; therefore, a priority is to identify the factors affecting intention, such as attitudes, subjective norms, perceived behavioral control, and self-efficacy [8, 10, 11]. In a previous study, the TPB model predicted the management of depression during and after pregnancy [12], and it could be used as a useful framework for predicting and explaining mental health behavior before pregnancy [13].

In addition to the components of the TPB, several studies have found that more severe depression affects the intention to manage depression [14–16]. Unlike previous related studies, this study was conducted with a focus on intentions for a healthy pregnancy. It is necessary to identify the current level of depression in college students with this new focus, including assessments of those who do not have depression; therefore, these results are expected to serve as the basis for planning programs to improve the intention of preventive depression management for a healthy pregnancy.

Recent studies have recommended prompt prevention and management of depression [17, 18]. However, most previous studies have emphasized interventions during or after pregnancy; even though there some studies have investigated the pre-pregnancy stage, preventive interventions for college students or adolescents have not yet been fully clarified [19]. These rapid interventions to prevent depression can have positive effects on a variety of health outcomes [3–6]. However, if the goal of it is set as a specific health outcome, the effect can be more pronounced. To the best of our knowledge, few studies have developed depression policies and interventions for reproductive health purposes. Therefore, as a basic investigation to confirm this, it is very novelty to identify depression prevention management intentions and related factors for college students who can promptly engage in interventions, and it will be helpful for future policy development based on the results of this study. Another weakness of existing studies is that most subjects of studies related to depression management for a healthy pregnancy were women [20]. Depression may be expressed differently in men and women according to their gender roles, and differences have been found in coping with depression (specifically, seeking counseling behavior) between men and women [3]. Traditionally, healthy pregnancy planning is often regarded as the domain and responsibility of women [21, 22]. Even recently, it has been found that young men's perceptions related to their preparation for pregnancy are still low [23]. Some studies have reported that male participation in preconception care improved maternal and newborn health [24]. Nonetheless, it is groundbreaking to investigate factors affecting the intention of preventive depression management for a healthy pregnancy among both women and men and to identify gender differences. This effort can contribute to health promotion for the next generation.

It is essential to take an approach that incorporates preventive and gender perspectives when preparing for a healthy pregnancy. This study aimed to determine the intention of preventive depression management focused on a healthy pregnancy among unmarried college students, as well as the factors influencing that intention, by applying the TPB model.

## Methods and materials

### Study design

This study had a cross-sectional descriptive design to explore the intentions of preventive depression management for a healthy pregnancy among unmarried college students who were not pregnant, as well as factors influencing those intentions, by applying the TPB model. In particular, this study was conducted to compare influencing factors by gender.

### Participants

A convenience sample was recruited. The potential participants were required to meet the following criteria: (a) age between 18 and 30 years; (b) college students; and (c) unmarried. Participants were excluded if they were: (a) currently preparing for pregnancy or (b) planning to become pregnant in the near future. These exclusion criteria were applied because this study aimed to determine the intention rather than the current behavior. In addition, since the average age at first marriage in Korea is over 30 [25], only those in their 20 s were recruited. The study participants were recruited from 21,279 students who were enrolled in the university, agreed to the informed consent form and responded to the online questionnaire. As a result, 862 people participated. Responses where demographic characteristics were not entered or responses were incomplete were excluded. The final sample for analysis consisted of 822 students (476 women and 346 men).

### Data collection

Data collection took place from July 20, 2019, to September 16, 2019. We delivered the online questionnaires to college students via an e-mail address registered at their school. The questionnaire took approximately 15–20 min to complete. At the end of the survey, the participants were provided a small gift (US $3) as compensation.

### Measures

#### General characteristics

Participants stated their age in years, gender, economic status (lower middle/upper middle), whether they had smoked in the previous month (yes/no), whether they had drunk alcohol in the previous month (yes/no), whether they had thought about suicide (yes/no), and whether they had sexual experience (yes/no). If they had sexual experience, participants were asked additional questions about how often they used contraception (never/sometimes/often/always) and whether they (or, in the case of men, their partners) had experienced pregnancy (yes/no). They also indicated whether they agreed that depression management during the preconception period is important for healthy pregnancy outcomes (strongly disagree/disagree/agree/strongly agree).

#### Depression scales

Depression was assessed using the Korean version of the Patient Health Questionnaire-9 (PHQ-9), a diagnostic measurement for depression with demonstrated validity for Korean college students [26]. The PHQ-9 comprises nine questions, each of which is answered on a 4-point scale ranging from 0 (not at all) to 3 (nearly daily) depending on the level of concern caused by symptoms of depression in the last 2 weeks. In addition, the 10th question rates the difficulty of daily life, although it is not included in the score. The total score ranges from 0 to 27, with higher scores indicating a greater level of depression. PHQ-9 scores of 0–4 are classified as normal, 5–9 as mild, 10–19 as moderate, and 20–27 as severe depression.

#### Constructs of the extended TPB

The extended TPB variables were measured with a newly developed TPB questionnaire, which was based upon guidelines for the construction of TPB questionnaires [27], with the addition of items to measure self-efficacy. This tool was developed to measure perceptions of depression management for a healthy pregnancy among unmarried college students. The extended TPB questionnaire contains five subscales assessing participants’ attitudes (4 items), subjective norms (4 items), perceived behavioral control (4 items), intention (6 items), and self-efficacy (5 items). Participants were asked to indicate their level of agreement with each question on a 5-point scale ranging from 1 (strongly disagree) to 5 (strongly agree). Attitude scores ranged from 4 to 20, and higher scores represented a more positive attitude towards preventive depression management for a healthy pregnancy. Subjective norm scores ranged from 4 to 20, and higher scores indicated more pressure from people around them. Scores for perceived behavioral control ranged from 4 to 20, and higher scores were interpreted as indicating the perception of having control over one’s own behavior. Intention scores ranged from 6 to 30, and higher scores indicated a higher intention of managing depression. For self-efficacy, the questions used a 4-point scale ranging from 1 (strongly disagree) to 4 (strongly agree), and higher scores indicated higher levels of self-efficacy. The 4-point scale was used because it prevented misuse of the midpoint [28]. For all questions, explanations are given that assume a situation where you are planning a future or present pregnancy.

### Ethical considerations

The questionnaire consisted of two main parts, and this survey was the second part of a larger project, the first study from which has already been published [29]. The goals of the larger project were to examine gender differences in the awareness of pregnancy and birth among college students. The project was approved by the Ethics Committee of Seoul National University. Prior to data collection, all participants were required to read the explanatory statement, which informed them that participation was completely voluntary, they could withdraw at any time, and completion of the questionnaire was considered to imply informed consent. Although the recruitment of research participants was conducted at the university where the researcher was working, there was no disadvantage to the research participants because they were anonymous.

### Data analysis

Data were analyzed using SPSS version 25 (IBM Corp., Armonk, NY, USA). Descriptive statistics (mean, standard deviation) were used to describe the general characteristics of the respondents. The chi-square test and t-test were used to compare gender differences in general characteristics, the current level of depression, and the constructs of the extended TPB. Hierarchical regression was performed to determine the factors influencing the intention of preventive depression management for a healthy pregnancy. Tests with a p-value less than 0.05 were considered statistically significant.

### Validity and reliability

This paper followed the STROBE guidelines (available at https://www.equator-network.org/reporting-guidelines/strobe/). All the researchers had received training in ethical principles for research. To minimize the threats of self-reported bias, data confidentiality and anonymity were guaranteed.

The depression questions used in this research were Korean-language versions, most of which had been tested in Koreans. For use in this study, after obtaining permission from the author, we obtained the original tools. The reliability of the Korean version of the PHQ-9 in the previous study [26] was confirmed by a Cronbach’s α value of 0.83, and the Cronbach’s α in the present study was 0.85.

The extended TPB variables were measured with a newly developed TPB questionnaire, which was based upon guidelines for the construction of TPB questionnaires [27], with the addition of items to measure self-efficacy. This tool was developed to measure perceptions of depression management for a healthy pregnancy among unmarried college students. A group of experts was selected to measure the content validity index (CVI). Each question was measured using a 4-point Likert scale, with responses from 'not at all reasonable' (1 point) to 'very reasonable' (4 points); the average score of each item was 3.4 points, yielding an average CVI for scale of 1.00. The reliability was confirmed by Cronbach’s α values of 0.66 (for attitudes), 0.78 (for subjective norms), 0.61 (for perceived behavioral control), 0.84 (for intention), and 0.74 (for self-efficacy), respectively. The total Cronbach’s α was 0.86.

## Results

### General characteristics of participants

A total of 822 students answered the questionnaire, of whom 476 were women and 346 were men. Table 1 compares general characteristics between genders. The mean age was 23.62 years (SD = 2.35) for men and 22.65 years (SD = 1.94) for women. The general characteristics that showed a statistically significant difference according to gender were age (t = 6.33, *p* < 0.001).

**Table 1:** General characteristics (N = 822)

Characteristics	Categories	Total	Women (n = 476)	Men (n = 346)	95% CI		χ^2^or t (p)
		Mean (SD) or n (%)			Lower	Upper	
Age (years)		23.06 (2.17)	22.65 (1.94)	23.62 (2.35)	0.67	1.28	6.33 (< 0.001)
	18–24 years	613 (74.6)	397 (83.4)	216 (62.4)			
	25–29 years	209 (25.4)	79 (16.6)	130 (37.6)			
Economic status	Lower middle	590 (71.8)	333 (70.0)	257 (74.3)			1.84 (0.174)
	Upper middle	232 (28.2)	143 (30.0)	89 (25.7)			
Smoking	Yes	89 (10.8)	36 (7.6)	53 (15.3)			12.48 (< 0.001)
Alcohol drinking	Yes	641 (78.0)	349 (73.3)	292 (84.4)			14.31 (< 0.001)
Have had thoughts about suicide	Yes	209 (25.4)	140 (29.4)	69 (19.9)			9.48 (0.002)
Sexual experience	Yes	452 (55.0)	226 (47.5)	226 (65.3)			25.76 (< 0.001)
Contraception use (n = 452)	Never	13 (2.9)	10 (4.4)	3 (1.3)			5.99 (0.112)
	Sometimes	25 (5.5)	14 (6.2)	11 (4.9)			
	Often	127 (28.1)	56 (24.8)	71 (31.6)			
	Always	287 (63.5)	146 (64.6)	141 (62.4)			
My (or, in the case of men, their partners) pregnancy experience (n = 452)	Yes	5 (1.1)	4 (1.8)	1 (0.4)			1.82^a^ (0.372
The importance of depression management during the preconception period for healthy pregnancy outcomes	Not at all important	2 (0.2)	1 (0.2)	1 (0.3)			1.49^a^ (0.685)
	Slightly important	16 (1.9)	7 (1.5)	9 (2.6)			
	Important	342 (41.6)	197 (41.4)	145 (41.9)			
	Very important	462 (56.2)	271 (56.9)	191 (55.2)			

^a^Fisher’s exact test*; SD* standard deviation; *CI* confidence interval

### Current level of depression among participants

Table 2 shows comparisons of current levels of depression by gender. There was no significant difference in total depression scores between men and women. In the subscales, women had a higher score for feeling down, depressed, or hopeless (t = − 2.86, *p* = 0.004), poor appetite or overeating (t = − 3.52, *p* < 0.001), and difficulty taking care of things at home, or getting along with other people (t = − 2.46, *p* = 0.014) than men. In contrast, men had a higher score for moving or speaking so slowly (t = 2.10, *p* = 0.036) than women. A slight majority (n = 429, 52.2%) of participants were classified as not having depression, while 280 (34.1%) had mild depression, 104 (12.7%) had moderate depression, and 9 (1.1%) had severe depression.

**Table 2:** Comparisons of current level of depression by gender (N = 822)

PHQ-9 Over the last 2 weeks, how often have you been bothered by any of the following problems?	Total	Women (n = 476)	Men (n = 346)	95% CI		χ^2^ or t (p)
	Mean (SD) or n (%)			Lower	Upper	
(not at all, 0; nearly every day, 3)						
1. Little interest or pleasure in doing things	0.72 (0.76)	0.74 (0.76)	0.69 (0.76)	− 0.15	0.06	− 0.82 (0.414)
2. Feeling down, depressed, or hopeless	0.72 (0.72)	0.78 (0.71)	0.63 (0.72)	− 0.24	− 0.05	− 2.86 (0.004)
3. Trouble falling or staying asleep, or sleeping too much	0.89 (0.88)	0.91 (0.90)	0.86 (0.87)	− 0.17	0.07	− 0.77 (0.439)
4. Feeling tired or having little energy	1.01 (0.83)	1.03 (0.82)	0.99 (0.85)	− 0.16	0.07	− 0.75 (0.455)
5. Poor appetite or overeating	0.70 (0.81)	0.78 (0.83)	0.58 (0.77)	− 0.31	− 0.09	− 3.52 (< 0.001)
6. Feeling bad about yourself—or that you are a failure or have let yourself or your family down	0.50 (0.78)	0.54 (0.80)	0.45 (0.76)	− 0.20	0.02	− 1.65 (0.100)
7. Trouble concentrating on things, such as reading the newspaper or watching television	0.37 (0.66)	0.40 (0.70)	0.34 (0.60)	− 0.15	0.03	− 1.23 (0.220)
8. Moving or speaking so slowly that other people could have noticed? Or the opposite—being so fidgety or restless that you have been moving around a lot more than usual	0.15 (0.47)	0.12 (0.43)	0.19 (0.52)	0.00	0.14	2.10 (0.036)
9. Thoughts that you would be better off dead or of hurting yourself in some way	0.14 (0.42)	0.13 (0.41)	0.15 (0.43)	− 0.03	0.09	0.92 (0.359)
Total score	5.20 (4.30)	5.42 (4.29)	4.89 (4.32)	− 1.12	0.07	− 1.74 (0.082)
10. If you checked off any problems, how difficult have these problems made it for you to do your work, take care of things at home, or get along with other people? (not difficult at all, 0; extremely difficult, 3)	0.52 (0.64)	0.57 (0.64)	0.46 (0.62)	− 0.20	− 0.02	− 2.46 (0.014)
Level of depression severity						
Normal (0–4)	429 (52.2)	237 (49.8)	192 (55.5)			3.63 (0.304) ^a^
Mild depression (5–9)	280 (34.1)	171 (35.9)	109 (31.5)			
Moderate depression (10–19)	104 (12.7)	64 (13.4)	40 (11.6)			
Severe depression (20–27)	9 (1.1)	4 (0.8)	5 (1.4)			

^a^Chi-square test; *SD* standard deviation; *CI* confidence interval

### Extended TPB variables related to preventive depression management for a healthy pregnancy

Comparisons of the TPB variables related to depression management for a healthy pregnancy according to gender are shown in Table 3. Male students had higher scores for subjective norms (t = 4.25, *p* < 0.001), perceived behavioral control (t = 8.40, *p* < 0.001), and intention (t = 2.36, *p* = 0.019) than female students.

**Table 3:** Theory of planned behavior variables and self-efficacy related to depression management during the preconception period for healthy pregnancy outcomes (N = 822)

For management of depression during the preconception period	Total	Women (n = 476)	Men (n = 346)	95% CI		t(p)
	Mean (SD)			Lower	Upper	
TPB variables (strongly disagree: 1, strongly agree: 5)						
Attitude	16.78 (2.02)	16.75 (1.97)	16.82 (2.08)	− 0.21	0.35	0.51 (0.613)
Subjective norms	14.26 (3.11)	13.87 (3.13)	14.79 (3.00)	0.50	1.35	4.25 (< 0.001)
Perceived behavioral control	12.88 (2.61)	12.25 (2.54)	13.74 (2.47)	1.14	1.84	8.40 (< 0.001)
Intentions	23.14 (3.47)	22.90 (3.34)	23.47 (3.62)	0.10	1.06	2.36 (0.019)
Self-efficacy (strongly disagree: 1, strongly agree: 4)	14.93 (2.28)	14.97 (2.23)	14.89 (2.33)	− 0.40	0.23	− 0.51 (0.614)

*SD* standard deviation; *CI* confidence interval

### Factors influencing the intention of preventive depression management for a healthy pregnancy

The hierarchical regression model for the intention of preventive depression management for a healthy pregnancy is presented in Table 4. In step 1, general characteristics that had gender differences, as shown in Table 1, were entered. The general characteristics with a significant influence on the intention of preventive depression management for a healthy pregnancy in men were drinking alcohol (*β* = − 0.13, *p* = 0.011) and the importance of depression management during the preconception period for healthy pregnancy outcomes (*β* = 0.40, *p* < 0.001). In step 2, attitudes, subjective norms, perceived behavioral control, and self-efficacy were added, and the analyses showed that step 2 predicted 36.0% in women and 43.0% in men of the intention, respectively. The significant factors of the final model in women were PHQ-9 score (*β* = 0.10, *p* = 0.014), the importance of depression management during the preconception period for healthy pregnancy outcomes (*β* = 0.08, *p* = 0.043), attitudes (*β* = 0.14, *p* = 0.001), subjective norms (*β* = 0.26, *p* < 0.001), perceived behavioral control (*β* = 0.10, *p* = 0.018), and self-efficacy (*β* = 0.34, *p* = 0.001). The corresponding factors in men were the importance of depression management during the preconception period for healthy pregnancy outcomes (*β* = 0.22, *p* < 0.001), attitude (*β* = 0.11, *p* = 0.023), subjective norms (*β* = 0.25, *p* < 0.001), and self-efficacy (*β* = 0.30, *p* < 0.001).

**Table 4:** Factors influencing the intention to manage depression during the preconception period (N = 822)

Independent variables	Women (n = 476)				Men (n = 346)			
	Model 1		Model 2		Model 1		Model 2	
	B	*β* (*p*)	B	*β* (*p*)	B	*β* (*p*)	B	*β* (*p*)
(Constant)	17.11		2.65		13.46		0.78	
Age	− 0.00	− 0.00 (0.967)	0.05	0.03 (0.482)	0.08	0.05 (0.320)	0.07	0.05 (0.277)
Smoking^†^	− 0.43	− 0.03 (0.941)	− 0.07	− 0.01 (0.877)	0.04	0.00 (0.910)	− 0.12	− 0.01 (0.774)
Drinking alcohol^†^	− 0.10	− 0.01 (0.946)	− 0.27	− 0.04 (0.352)	− 1.27	− 0.13 (0.011)	− 0.68	− 0.07 (0.109)
Having thought about suicide^†^	0.01	0.00 (0.885)	0.18	0.02 (0.542)	− 0.51	− 0.06 (0.267)	− 0.05	− 0.01 (0.904)
Sexual experience^†^	0.30	0.05 (0.858)	0.12	0.02 (0.646)	0.46	0.06 (0.258)	0.09	0.01 (0.787)
PHQ-9 score	0.00	0.00 (0.884)	0.08	0.10 (0.014)	0.01	0.01 (0.842)	0.06	0.07 (0.117)
The importance of depression management during the preconception period for healthy pregnancy outcomes	1.64	0.26 (0.985)	0.50	0.08 (0.043)	2.53	0.40 (< 0.001)	1.38	0.22 (< 0.001)
Attitudes			0.24	0.14 (.001)			0.19	0.11 (.023)
Subjective norms			0.28	0.26 (< 0.001)			0.30	0.25 (< 0.001)
Perceived behavioral control			0.13	0.10 (0.018)			0.13	0.09 (0.065)
Self-efficacy			0.51	0.34 (0.001)			0.47	0.30 (< 0.001)
R^2^ (ΔR^2^)	0.07		0.37 (0.30)		0.19		0.44 (0.25)	
*Adj* R^2^	0.06		0.36		0.18		0.43	
F (*p*)	5.23 (< 0.001)		25.11 (< 0.001)		11.43 (< 0.001)		24.28 (< 0.001)	

^†^Dummy variables (Ref. no); Adj R^2^ adjusted r square

## Discussion

Early adulthood is a significant period for preventive depression management for a healthy pregnancy, but early identification of adults needing depression management is a major challenge. The definition of the period of preparation for pregnancy has been redefined in recent studies. A novel concept analysis study of preconception included the public health perspective of conception as not involving a conscious decision [2]. The World Health Organization (WHO) also considered preconception as involving the provision of preventive, promotive, or curative health and social interventions before conception occurs, and therefore emphasized the need for a wide range of interventions according to this definition [30]. However, most preconception studies have been conducted in people with the intention to become pregnant [31–33], and these existing limited studies described hindrances towards a healthy pregnancy. This study makes the suggestion that preparing for a healthy pregnancy involves prevention.

Another important aspect of encouraging preparations for a mentally healthy pregnancy is the need to recognize that both men and women are target populations [2, 30]. In the present study, the intention of preventive depression management for a healthy pregnancy was found to be higher in men than in women among unmarried college students. This result closely corresponds with those found in recent earlier studies that reported gender differences in intentions to prepare for a healthy pregnancy [34]. The reason for this is that men also showed higher subjective norms and perceived behavioral control than women in our study, and these variables influence intention according to the TPB model [8]. Therefore, these factors should be considered more carefully for women. Although intention showed a statistically significant difference between men and women, both were above the middle. Some existing studies have provided evidence to support regular encouragement for men regarding the healthy management of pregnancy [35, 36]. Based on this evidence, public health professionals can expect that individuals of both genders can be encouraged to manage depression for a healthy pregnancy by providing appropriate interventions urging them to continue to maintain their intention. In this study, unmarried college students showed that they were capable of preparing for pregnancy in their current environment, because they did not have low intentions for preparing for a healthy pregnancy.

The hierarchical regression analysis conducted in this study showed that the explanatory power increased significantly when the TPB constructs were added to the general characteristics. This result aligns with the findings of other studies that TPB constructs can explain the intention to seek mental health treatment [37–39]. Of the predictor variables related to the expanded TPB model, self-efficacy was found to be the most effective. Self-efficacy reflects the concept that one is confident of being able to manage depression for a healthy pregnancy [40]. This study confirmed that the extended TPB model is a promising tool to successfully address improving that intention for both genders, especially by reinforcing self-efficacy.

This study found that the current level of depression in women affected their intention. This finding supports a previous study, according to which depression severity and perceived necessity for depression treatment were predictors of intention to seek mental healthcare [41]. A study applying the TPB model confirmed that psychological symptom severity directly increased the intention for help-seeking behavior [16]. Therefore, understanding the degree and difference between levels of depression in current college students is important for suggesting intervention strategies. In these participants of men, depression did not affect intentions differently from women. These findings suggest that it is especially important to identify the level of depression in women and to recognize the need for management to prevent future depression during pregnancy.

Variables for certain general characteristics entered into the model such as age, sexual experience, history of smoking or drinking alcohol, and thoughts about suicide were not predictors of intention. This study was conducted at one university, it would be difficult to generalize these findings to all unmarried college students. These results should be interpreted cautiously; although the aforementioned variables were not shown as important in the model, it remains possible that these variables are relevant for explaining the intention of preventive depression management for a healthy pregnancy. Since the study was conducted among people who did not intend to become pregnant yet, there may be differences in the relationship between existing research results regarding health behavior, preventive depression behavior, or intentions [32, 33]. Several studies have suggested that these general characteristics, including smoking and/or alcohol experience, affect depression [32, 33], and this study also found that depression affected intention. Therefore, more research remains to be done on the relationship between these health behavior factors and the intention of preventive depression management for a healthy pregnancy through a longitudinal study.

The present study presents feasible and applicable results. First, this study is one of the first studies to confirm the recently developed concept of preconception as a period for early interventions. In this context, it is necessary to collect evidence regarding entry into a healthy pregnancy by confirming these findings through studies on diverse health promotion topics and age groups, including adolescents. In addition, this study also investigated men’s perceptions of preparing for a healthy pregnancy, unlike traditional female-oriented research. Our study results provide evidence with university health clinic nurses and/or community psychiatric nurses regarding the need for preventive interventions and directions to develop interventions to improve the intention of depression management among unmarried college students. In particular, based on the results of this study, it is necessary to intervene with all the constructs of the TPB model, with a particular focus on self-efficacy as an element of preventive interventions for both men and women. It is also important to check the current depression status of unmarried female college students and to address depression preferentially in high-risk groups.

This study has some limitations. First, the sample was recruited from only one college in Korea. In addition, an existing meta-analysis of the online survey responses found that the minimum value was 5.0% [42]. Our results are similar to the previous study, but it was still low, at 4.1%. When comparing the results of a national-level survey in Korea for subjects aged 19–29 with the general characteristics of this study subjects, the smoking rate and the drinking rate showed differences of 13.9% and 9.7%, respectively [43]. Thus, the responses cannot be generalized to all unmarried college students. A second limitation is that because we used the TPB model, we did not consider other individual predisposing factors influencing the intention of preventive depression management. According to previous studies, knowledge, skills, past help-seeking experience, and confidence in the formation of new social relationships may affect this intention and behavior [3, 4]. Therefore, caution is needed in interpreting these results, as these variables, which were not evaluated in this study, may interact with the variables of TPB. Additionally, this study’s subjects responded with the assumption that they were planning a future or present pregnancy. A third limitation, however, was that the study design did not reflect the perceived pregnancy planning potential differently among individuals. It is also difficult to know how well the hypothesized pregnancy plan matches the actual pregnancy planning situation. In addition, this study used reproductive health indicators limited to sexual experience and contraceptive use. Considering more parameters related to reproductive health could be a much more desirable study. Last, the limitation is that this cross-sectional study only examined intention and did not assess actual behavior. Intentional-behavioral correlations are usually, but not always, robust [39]. Furthermore, since this is a cross-sectional study, the temporal order of the variables cannot be determined, and the direction of the causal relationship is unclear. Therefore, additional research should use a longitudinal design to confirm the extent to which intentions translate into actual depression management behavior for a healthy pregnancy and to verify the associations shown in this study.

## Conclusions

The present study demonstrated that the factors affecting the intention of preventive depression management for a healthy pregnancy include components of TPB in both women and men, of which self-efficacy had the greatest effect. Current depression and perceived behavioral control were statistically significant only for women. These findings could help health professionals, including nurses, recognize gender differences and common characteristics for the intention of preventive depression management for a healthy pregnancy, and provide evidence to develop and implement interventions. These efforts will ultimately reduce high-risk pregnancies due to poor mental health.

## Data Availability

The datasets generated during and/or analyzed during the current study are not publicly available due to protection and confidentiality, but may be available from the corresponding author on reasonable request.

## Abbreviations

CDC: Center for Disease Control and prevention
CI: Confidence interval
CVI: Content Validity Index
IBM: International Business Machines corporation
NY: New York
OECD: Organisation for Economic Co-operation and Development
PHQ: Patient Health Questionnaire
SD: Standard deviation
SPSS: Statistical Package for Social Sciences
STROBE: STrengthening the Reporting of OBservational studies in Epidemiology
TPB: Theory of planned behavior
US: United States
USA: United States of America
WHO: World Health Organization
